# Pattern of use of contact lens among college students: A cross-sectional study in coastal Karnataka

**DOI:** 10.4103/0301-4738.57159

**Published:** 2009

**Authors:** B Unnikrishnan, Shakir Hussain

**Affiliations:** Department of Community Medicine, Kasturba Medical College, Mangalore, India. (Affiliated to Manipal University)

**Keywords:** Contact lens, India, Karnataka, students

## Abstract

The use of contact lens (CL) for the correction of refractive errors, cosmetic use and their usage as a therapeutic modality for corneal pathologies has increased tremendously over the years. The present study was conducted with the aim to find a pattern of CL use amongst college students with a focus on the rationale for CL use and problems related to their use. This study includes 371 college students who were current users of CL at the time of the study. Results showed that 96.8% of the CL users use the ‘daily wear type’ of CL. Most quoted reasons of usage were comfort and convenience (61.2%) with cosmetic benefit (42.9%) as the next most common reply. Common complaints were that of general discomfort (foreign body sensation), dry eyes and watering eyes. Educated use of CLs amongst its users is advised in view of the symptoms and associated complications that may occur.

Popularity of contact lens (CL) continues to increase with regular improvement in materials and variants suitable for a variety of users.[1] The ideal CL for refractive errors has proved difficult to find with reports of complications with even the most advanced systems available.[2–4] Recent studies have shown the use of CL for refractive error correction to be higher and more common among the younger strata of population.[5–7] Complications most commonly associated with use include dry eye, giant papillary conjunctivitis, corneal abrasion, corneal edema, corneal ulcer, keratitis and neovascularization.[8] The awareness of these complications was found lacking amongst the younger users and 87% of these users preferred CL use in spite of the ocular problems related to their use.[9] Cosmetic benefits and convenience were the most common reasons cited for CL use.[5,6] Knowledge about use pattern would prove useful to general ophthalmologists and optometrists in guiding young prospective users in lens type, hygiene and pattern of use. The aim of this study was to find a pattern of CL use amongst college students with a focus on the rationale for CL use and problems related to their use.

## Materials and Methods

The present cross-sectional study was carried out in 18 colleges of coastal Karnataka comprising two districts, namely Dakshina Kannada (Mangalore) and Udupi.

The sample size was calculated based on the previous pilot study in which 60% of the CL users had symptoms associated with CL use, the minimum sample size was found to be 369 CL users with precision of 5%, power of 80% and 95% confidence interval. The study population consisted of 6850 college students from 18 different colleges in coastal Karnataka. These students were surveyed about their CL use, out of which 392 students were currently using CLs, 21 students declined to participate in the study and the final study sample size came to 371 college students.

Data was collected by using a “pre-tested” semi-structured questionnaire. The questionnaire consisted of 22 items. It elicited demographic profile, pattern of use of CLs and problems related to its usage. The questionnaire was pretested among a group of 20 college students in Mangalore, Karnataka, and was revised to enhance its clarity and comprehension. Before the start of the study the investigator visited the various colleges and obtained the permission from the authorities of the colleges. Data were collected by personal interview, after obtaining informed consent from the study subjects. The data were analyzed using SPSS Version 11 statistical test, Chi square for association was used and P<0.05 was considered as statistically significant.

## Results

Out of 6850 college students from 18 different colleges of coastal Karnataka, 392 students were found to be current users of CL, 94.6% (371) students consented to participate in the study. Of the total surveyed 79.5% (295) were females. A majority of the CL users 92.5% (343) were in the age group of 17-22 years. In lens type preference 96.8% (359) were found to use daily wear type lens, 1.6% (six) extended wear-soft type and the rest 1.6% (six) used hard/rigid gas-permeable lens (RGP). All users of RGP lens stated that they have keratoconus.

Hours of daily wear and problems associated with use are provided in Table 1. The association between hours of daily wear and problems due to CL use was found to be statistically significant (*P*=0.04)

**Table 1:** Hours of daily wear vs. problems related to use of contact lens among the respondents

Hours of daily wear	Problems related to use of contact lens				Total
		
	YES		NO		
			
	No	%	No	%	
< 8 h	62	88.6	8	11.4	70
8 − 16 h	201	75.0	67	25.0	268
> 16 h	24	72.7	9	27.3	33
Total	287	77.4	84	22.6	371

Chi square = 6.28 P = 0.043

In reasons for using CLs, it was found that 61% (227) of the respondents quoted comfort and convenience as the most common reason for CL use, followed by cosmetic reasons 43% (159), clear, wider field of vision and sports 11.6% (43), keeping the eye power stable 5.9% (22) and keratoconus 1.3% (five).

Among problems related to the use of CL, it was found that 47.7% (177) of the respondents quoted general discomfort, followed by dry eyes 38% (141), watering eyes 31.5% (117), allergies to solution 20.2% (75) and red eyes 19.4% (72) as the problems faced due to CL use. Other problems (poor near vision, poor distant vision, crusting on eyelids, short wearing time, frequent CL deposits) were quoted by 24.8% (92) of the respondents; around 20.7% (77) of the respondents had no problems associated with the use of CL.

## Discussion

In the present study the majority of the respondents preferred daily wear CLs which is consistent with the result of a similar study done in Singapore.[6] In our study 79.9% of the CL users were found to be females, the same gender predilection to CL use was found in two other studies.[5–7]

Convenience, comfort and cosmetic purpose were cited as the main reason for CL use and keratoconus was cited as a reason by 1.3% of the CL users. All RGP lens users in the study stated that they have keratoconus. These findings are consistent with those from other studies.[10]

Hours of daily wear was also found to be significantly associated with problems related to CL use. It was found that the CL users using CL for ≤8 h a day were more prone to problems than individuals using for more than 8 h a day. It is known that CL use alters corneal physiology hence longer hours of daily use elicits more symptoms. This finding, though contradictory may be confounded by the fact that the short hours of use in a day may be due to symptoms relating to CL usage. The association of duration of CL use and problems related to use was found to be statistically significant.

The symptoms reported with CL use are seen with almost all new users but tend to come down as the users get accustomed to CL. Such symptoms may also be associated with factors like weather conditions, dusty environments, and general use hygiene.[8]

A clinical examination of the respondents would have been appropriate but was not possible due to fund constraints. Respondents ranged from those using for a few months to many years which might confound the results.

In spite of improvement in design and materials of CL, problems associated with its use continue to persist, as 79.3% of the CL users in our study experienced problems related to its use. This knowledge would prove useful in guiding young prospective CL users in terms of lens type and hygiene.

## APPENDIX

**CONTACT LENS USE PATTERN AMONGST COLLEGE STUDENTS: A CROSS-SECTIONAL STUDY**

This questionnaire is designed to help us better evaluate your contact lens (CL) needs. Please answer all questions as completely as possible. Thank you for your time. Please note that all personal information shall be kept confidential.

**Table:**

Respondent' Name:	Age:	Sex:	
Address:	College and Course:		
	Date:		
Please tick the appropriate option/specify as required:			
1. Do you currently wear contact lenses (CL)?		□ YES	□ NO
2. Type of lens		□ Soft-extended wear	
		□ Soft-disposable	
		□ Rigid gas-permeable	
3. How often do you replace them?			
4. How old are your current CL?			
5. How many hours a day on an average do you wear CL?			
6. How many days a week do you wear CL?			
7. How many years ago did you began wearing CL?			
8. Do you sleep with CL on?		□ YES If Yes (How often?)----------	
		□ NO	
9. a. what brand of CL solution do you use?			
b. do you clean after each wear in		□ the evening or in	□ the morning
c. how often do you use anti-protein tablets?			
10. How many times have you visited your Opthalmologist after starting to use CL?			
11. If you are not currently wearing CL, have you ever worn or tried to wear CL in the past?		□ YES	□ NO
		If Yes how long and what type of CL?	
12. Why did you stop wearing them?			
13. Have you had any eye infections/problems related to CL wear?		□ YES	□ NO
		If yes please specify in #17	
14. Do you have any systemic allergies or asthma?		□ YES	□ NO
15. Have you had problems with CL use in a dusty environment or with fumes?		□ YES	□ NO
16. How would you describe your desire to wear CL?		□ MILD	
		□ MODERATE	□ HIGH
17. Please check if you have had any of the conditions or symptoms from wearing CL?		□ Dry eyes	□ Red eyes
		□ Watering eyes	□ Discomfort
		□ Poor near vision	□ Poor distant vision
		□ Crusting on eyelids	
		□ Short wearing time	
		□ Allergies to solution	
		□ Frequent CL deposits	
		If any others, specify	
Do you			
1. Currently have prescription eyewear?		□ YES	□ NO
		If yes, specify power	Right eye: Left eye:
2. Spend a lot of time outdoors?		□ YES	□ NO
3. Currently have prescription sun wear?		□ YES	□ NO
4. Use a computer on a daily basis? Hours/day? (Specify) ________			
5. Have more than one pair of current eyewear?		□ YES	□ NO
6. Have visual difficulty when driving?		□ YES	□ NO
Why do you prefer contact lenses? ________________________________________			
